# The Heterogenous Effect of COVID-19 on Liver Transplantation Activity and Waitlist Mortality in the United States

**DOI:** 10.3389/fsurg.2021.669129

**Published:** 2021-05-18

**Authors:** Qing Yuan, Omar Haque, Taylor M. Coe, James F. Markmann

**Affiliations:** ^1^Department of Urology, Chinese PLA General Hospital, Beijing, China; ^2^Center for Transplantation Sciences, Department of Surgery, Massachusetts General Hospital, Boston, MA, United States; ^3^Harvard Medical School, Boston, MA, United States; ^4^Department of Surgery, Beth Israel Deaconess Medical Center and Harvard Medical School, Boston, MA, United States; ^5^Center for Engineering in Medicine and Surgery, Massachusetts General Hospital and Harvard Medical School, Boston, MA, United States; ^6^Shriners Hospitals for Children, Boston, MA, United States

**Keywords:** COVID-19, liver transplant, waitlist mortality, organ allocation, united network for organ sharing

## Abstract

**Background:** The COVID-19 pandemic curtailed the practice of liver transplantation (LT), which lacks a temporizing life-saving measure for candidates on the waitlist.

**Aims/Objectives:** The objective of this research was to (1) determine the effect of decreased LT activity on waitlist mortality in the United States and (2) assess if this effect was homogenous across the country.

**Methods:** We conducted a retrospective, cross-sectional analysis utilizing United Network for Organ Sharing (UNOS) data assessing 3,600 liver transplants from January 1, 2020 to June 2, 2020. COVID-19 incidence data was taken directly from the New York Times case count.

**Results:** During weeks 10 to 15 of 2020, there was a 38% reduction in the number of LTs performed nationally, which was temporally associated with a transient 97% increase in waitlist mortality. When stratified by UNOS region, waitlist mortality was inversely correlated with the number of LTs performed in all 11 regions. However, the range of the association strength (r) was large (Pearson correlation coefficient range: −0.73 to −0.01).

**Conclusion:** Interruptions in LT activity due to COVID-19 were associated with rapid increases in waitlist mortality, and these effects were unevenly distributed among candidates across the United States. The transplant community can utilize these results to mitigate inequalities in transplant allocation between UNOS regions and advocate for the uninterrupted practice of LT should another pandemic surge or COVID-19 variant arise.

## Introduction

On March 11, 2020, the World Health Organization declared COVID-19 a global pandemic, and the ramifications of the virus had a complex impact on health care delivery across the United States and the world. In an attempt to preserve resources for a surge of COVID-19 patients requiring intensive care unit (ICU) care and ventilator support, elective surgical cases were ordered to be stopped by state and local governments. The handling of life-saving urgent surgeries varied depending on the nature of the case and resource availability (1, 2).

Thus, the impact of COVID-19 on solid organ transplantation was multifactorial. Transplant centers had to balance the critical needs of patients on the organ transplant waitlist awaiting vital organs with the personnel and ICU capacities of hospitals (3, 4). These decisions were especially critical in the field of liver transplantation, which lacks an alternative life sustaining therapy comparable to what dialysis provides for those with renal failure (2). Furthermore, COVID-19 created issues regarding inadequate donor testing at some centers, travel bans between various regions, and the potentially deleterious consequences of immunosuppressed liver transplant (LT) recipients recovering in COVID-19 positive ICUs (5–7).

In Europe, the crude death rate from COVID-19 was 7% higher for LT recipients compared to the general population. Accordingly, 86% of LT centers in Europe reported testing all donors for COVID-19 and 91% of centers did not transplant organs from COVID-positive donors. In the occurrence of a COVID-positive LT candidate, 55% of centers in Europe canceled the transplant and 34% of centers reallocated the donor organ to another recipient (1).

In the United States, 67.7% of living donor LT centers (21 out of 31) completely suspended live donor liver transplantation and 73.3% of all deceased donor LT centers (44 out of 60) reported implementing restrictions to their clinical activity. In addition, 85.4% of United States LT centers adopted donor testing protocols (5). These changes in LT practice reflected the altered risks and benefits of conducting LTs in the COVID-19 era, especially considering the associated risks and hospital personnel required to care for newly immunocompromised LT recipients in a COVID-rich environment (8).

We hypothesized that the overwhelming healthcare burden of COVID-19 in the United States reduced the number of LTs conducted during the pandemic, with ramifications concerning patients waiting for a LT. We aimed to quantify this decrease and assess its correlation with LT waitlist mortality. The United States was unique in its varied response to the COVID-19 pandemic based on individual state responses to testing and social distancing. Additionally, the United States is divided into 11 distinct United Network for Organ Sharing (UNOS) regions (9). Thus, the second objective of this work was to assess the regional variation in liver transplantation and LT waitlist mortality based on regional COVID-19 incidence rates.

## Methods

### Data Sources

This retrospective, cross-sectional study analyzed data from the Organ Procurement and Transplantation Network (OPTN) STAR file release on July 2020 based on data collected through June 2020. The content in this paper is the responsibility of the authors alone and does not necessarily reflect the views or policies of the Department of Health and Human Services, nor mentions trade names, commercial products, or organizations imply endorsement by the United States Government. COVID-19 incidence data was taken directly from the New York Times updated United States case count (10).

### Study Population and Outcomes

We identified all deceased donors who had at least one organ donated, and 3,600 LTs (3,416 adult and 184 pediatric LTs) from January 1, 2020 to June 2, 2020 in the United States. Outcomes were number of liver transplants performed per week (stratified by adult vs. pediatric and by UNOS region), waitlist mortality per week, number of deceased donors in 2020 (stratified by donation after circulatory death vs. donation after brain death), and reasons LT candidates were removed from the waitlist.

### Statistical Analysis

Trends of LTs and newly infected COVID-19 cases were presented as absolute numbers by week since January 1, 2020. Weeks 10 and 15 of 2020 were noted as the inflection points in the incidence of COVID-19 in the United States. Incidence was calculated from the number of new COVID-19 cases per week. Waitlist mortality was calculated from the number of LT candidates who reportedly died divided by the number of total candidates on the waitlist during that week. All analyses including Pearson correlation coefficients (*r*) and coefficients of determination (*r*^2^) were performed using RStudio software, version 1.1.456 (R. RStudio, Inc., Boston, MA, USA).

## Results

**Figure 1:**
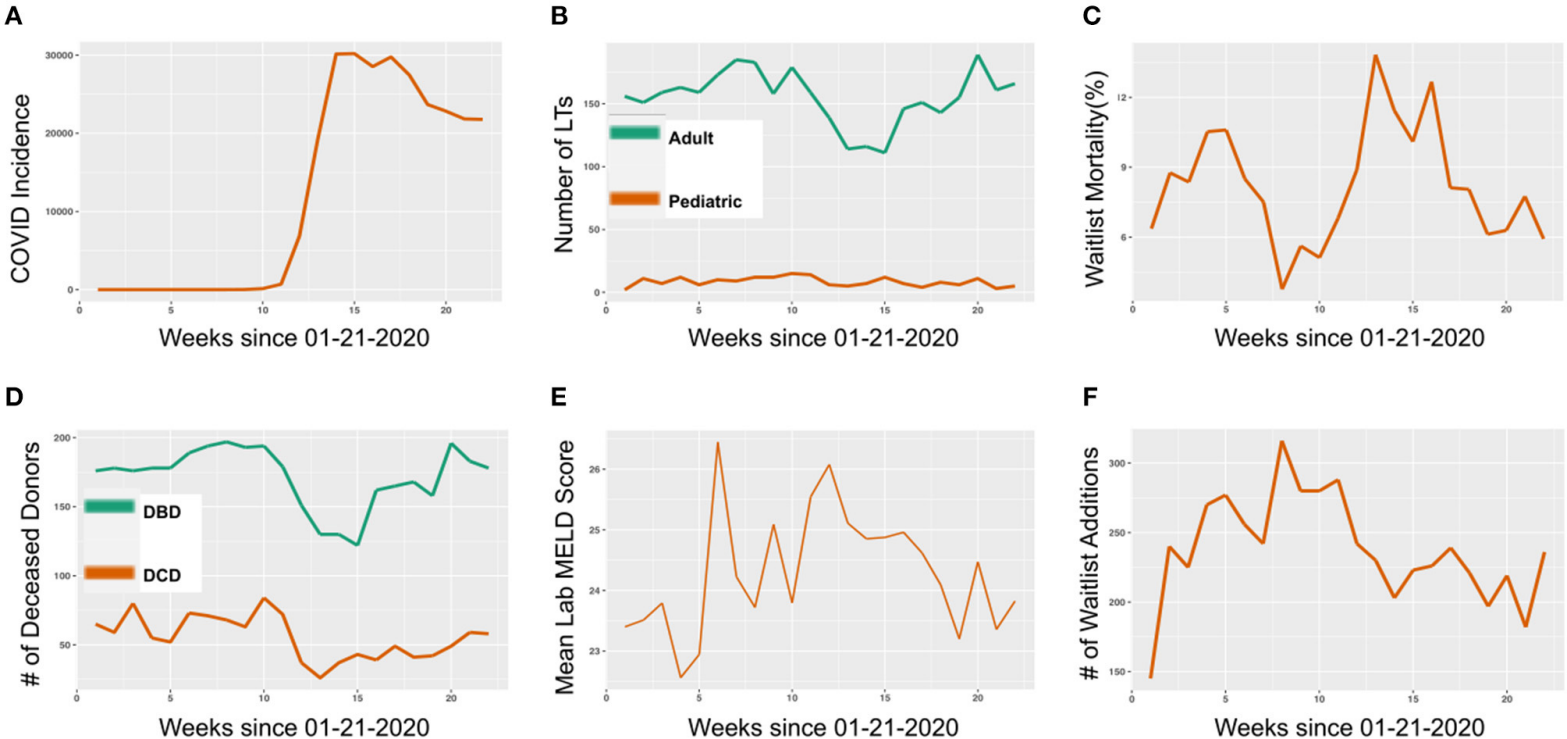
National United States liver transplant (LT) trends in 2020. **(A)** COVID-19 incidence by week in 2020 (Incidence defined as number of new cases per week). **(B)** Number of LTs performed by week in 2020, stratified by adult and pediatric LTs. **(C)** Candidate LT waitlist mortality by week in 2020. **(D)** Number of deceased organ donors, stratified by donation after brain death (DBD) and donation after circulatory death (DCD) donation, by week in 2020. **(E)** Mean lab MELD score of LT recipients by week in 2020. **(F)** Number of new LT waitlist additions by week in 2020.

The incidence of COVID-19 began to rise exponentially in the United States by the 10th week of 2020. Between the 10th and 15th week of 2020, the incidence of COVID increased from 128 to 30,188 cases/week (Figure 1A). The number of adult LTs performed per week decreased from 179 in week 10 to 111 in week 15, a 38% decrease, while the number of pediatric LTs remained constant between 12 and 15 per week (Figure 1B). The decrease in adult LTs was accompanied by a 97% rise in LT waitlist mortality, from 5.1% in week 10 to 10.1% in week 15 (Figure 1C). On a national level, the decrease in deceased donors during the COVID-19 surge paralleled the number of LTs performed. From weeks 10 to 15, the number of donation after brain death (DBD) donors decreased by 37%, from 194 to 122 donors/week. The percent decrease in number of deceased after circulatory death (DCD) donors was 48%, from 84 to 43 donors/week (Figure 1D). During this time, the mean lab model for end-stage liver disease (MELD) score for recipients only rose by 4.62% (from 23.8 in week 10 to 24.9 in week 15) (Figure 1E). Finally, the pandemic surge led to a 20.4% decline in number of newly listed patients (from 280 in week 10 to 223 in week 15) (Figure 1F).

**Figure 2:**
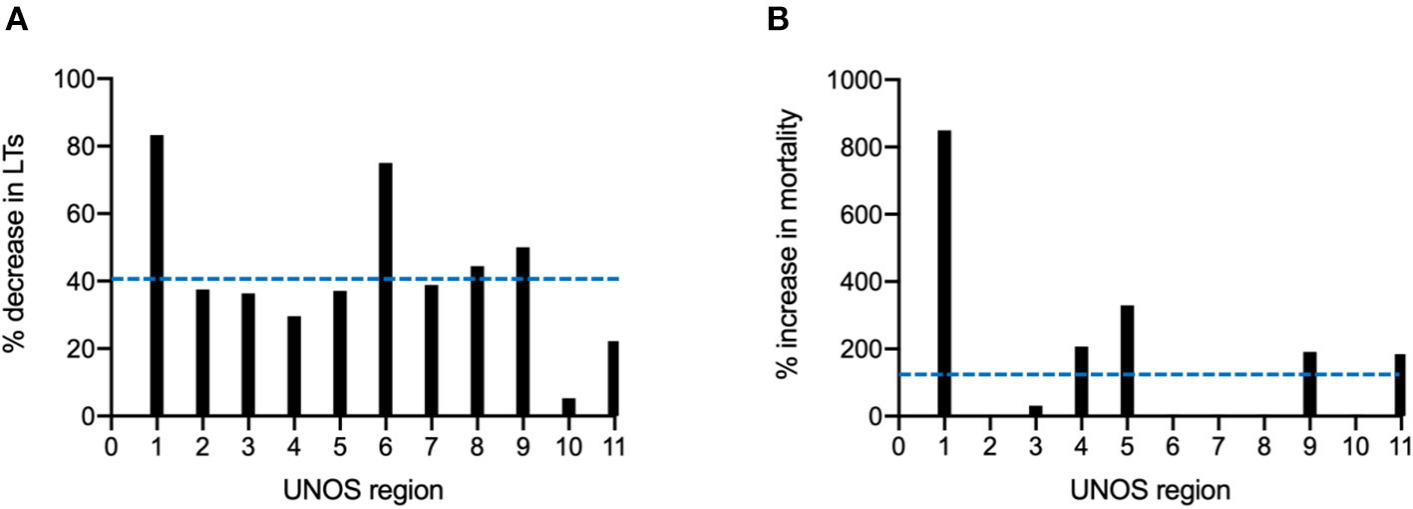
Regional variation in liver transplantation during the COIVD-19 pandemic in the United States. **(A)** Percent decrease in LTs performed from week 10 to 15 of 2020, stratified by the United Network for Organ Sharing (UNOS) region. **(B)** Percent increase in candidate LT waitlist mortality from week 10 to 15 of 2020, stratified by UNOS region. Dashed blue lines represent means across all 11 UNOS regions.

Due to the varied timing of the COVID peak in different areas of the country, and the heterogenous national response to COVID-19, there was considerable variation in transplant center activity during the initial surge of the pandemic (weeks 10 to 15 of 2020). During these weeks, all UNOS regions exhibited a decrease in the number of LTs performed per week. However, the range was large; region 1 had a 83% decrease in LTs performed per week while region 10 only had a 5.3% decrease. The average decrease in LTs performed per week by UNOS region was 41.8% (Figure 2A). LT waitlist mortality also exhibited high variation around the country. Region 1 saw a 850% increase in LT waitlist mortality (from 0.05 to 0.5%) from week 10 to 15 while some UNOS regions in the Midwest (6, 7, 8, and 10) had no decrease in mortality over the same period. The average increase in LT waitlist mortality from week 10 to 15 was 134% (Figure 2B).

**Figure 3:**
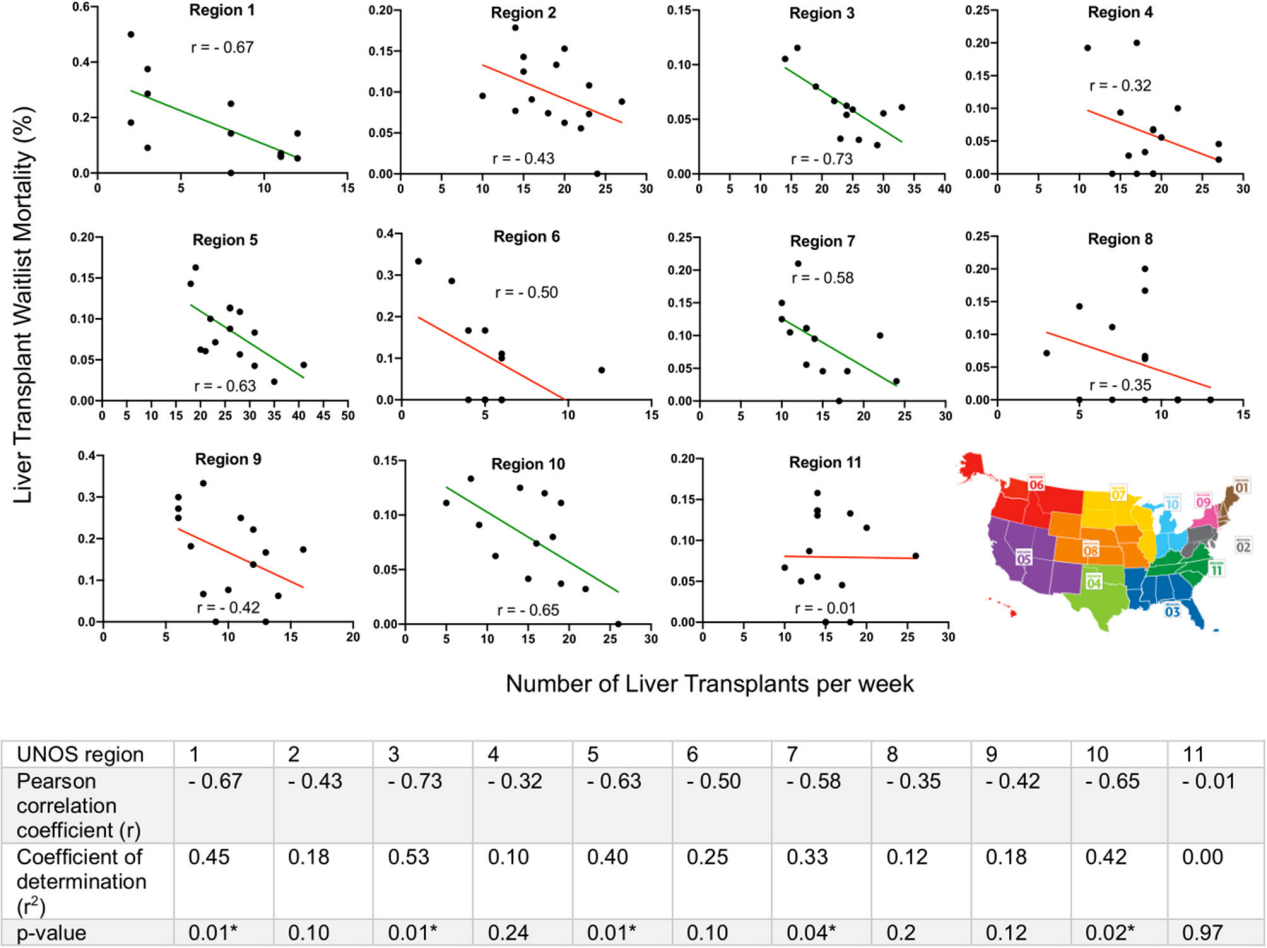
Regional variation in liver transplant (LT) activity and waitlist mortality in the Unites States during COVID-19. Pearson correlation coefficients (*r*) assessed the strength of association between the number of LTs performed in the first 15 weeks of 2020 and LT waitlist mortality, stratified by United Network for Organ Sharing (UNOS) region. All 11 UNOS regions exhibited a negative association, however, there was a large amount of regional variation (r range −0.73 to −0.01). Green lines indicated statistically significant negative associations, red lines indicated lack of statistical significance. Coefficients of determination (*r*^2^) calculated from the square of the Pearson correlation coefficient. *Significance level *p* < 0.05.

Pearson correlation coefficients (*r*) assessing the association between number of LTs performed during the first 15 weeks of 2020 and LT waitlist mortality were calculated for all 11 UNOS regions. UNOS regions 1, 3, 5, 7, and 10 all showed statistically significant negative associations (negative r) between the number of LTs performed and waitlist mortality; thus, as the number of liver transplants decreased during the height of the pandemic, the LT waitlist mortality increased. UNOS regions 2, 4, 6, 8, 9, and 11 also had negative Pearson correlation coefficients (*r*) but did not have statistically significant associations between number of LTs performed and waitlist mortality. Coefficients of determination (*r*^2^) indicated what percent of the variability of LT waitlist mortality was due to decreases in number of LTs conducted. Region 1, 3, 5, and 10 had the highest *r*^2^ at 45%, 53%, 40%, and 42% respectively while region 11 had the lowest at 0%. Finally, there was a large amount of regional variation in the strength of association between LTs performed and LT waitlist mortality (r range −0.73 to −0.01) (Figure 3). These changes in waitlist mortality during COVID-19 were also not secondary to sicker LT candidates with higher MELD scores, as there was no correlation between a region's average MELD score change and LT waitlist mortality shift during weeks 10 to 15 of 2020 for all UNOS regions (*r* = 0.05, *p*-value 0.87) (Supplementary Figure 1).

**Figure 4:**
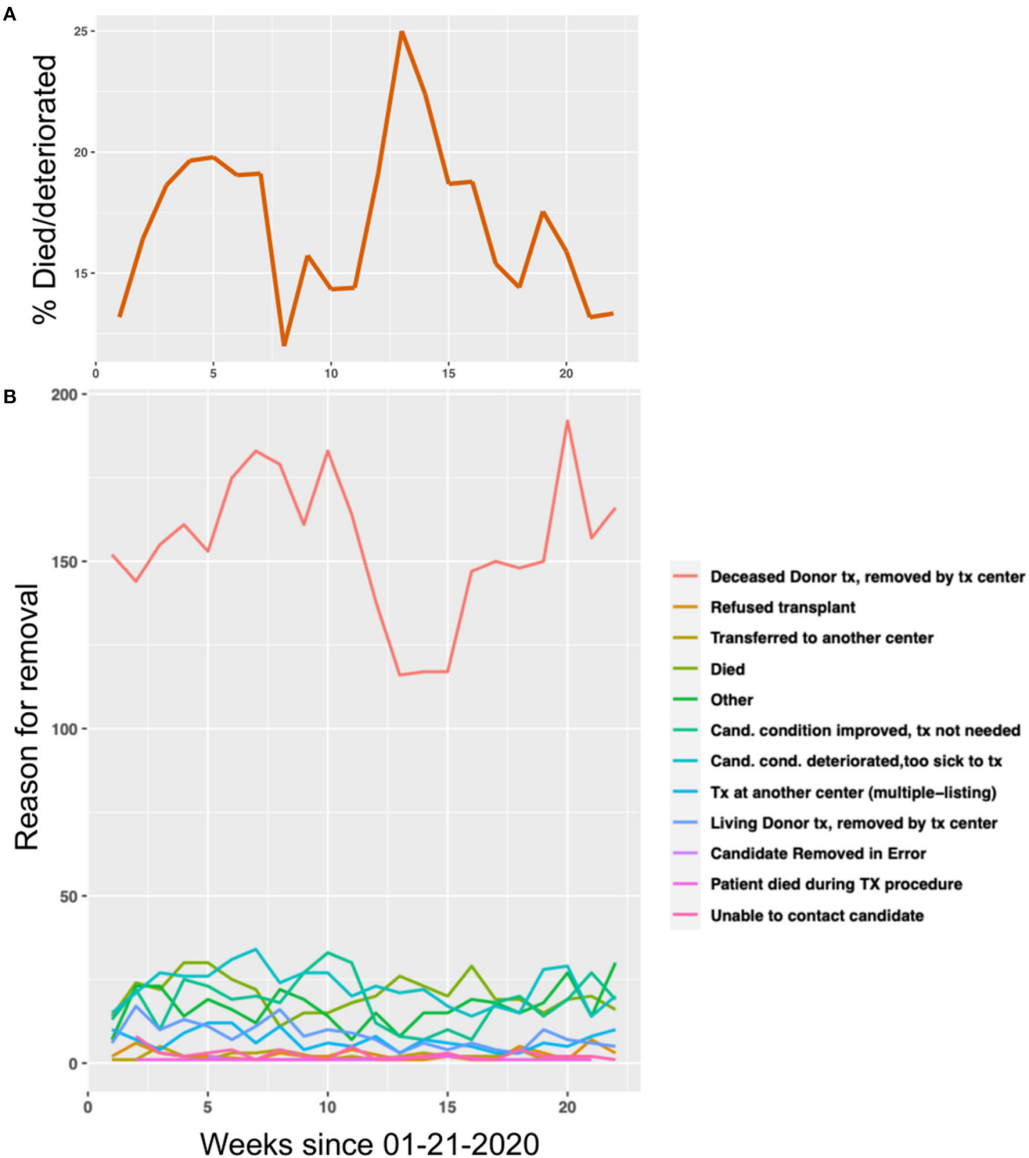
The effect of the COVID-19 pandemic on liver transplant (LT) waitlist activity. **(A)** The percent of people in the United States who died or clinically deteriorated on the LT waitlist in 2020, by week. **(B)** The number of people removed from the LT waitlist in the United States, stratified by reason for removal, in 2020 by week.

The percent of candidates who died or clinically deteriorated on the liver transplant waitlist at the national level increased from 15 to 25% between weeks 10 and 13 of 2020 (Figure 4A). While the majority of the reasons for removal from the LT waitlist remained constant during the COVID-19 pandemic, there was a temporal relationship between the rapid increase in percent of candidates who died or clinically deteriorated at week 13 and the number of deceased donor LTs performed at that time (Figure 4B).

## Discussion

In this analysis, we quantified the impact of the COVID-19 pandemic on LT activity and waitlist mortality in the United States. We demonstrated a temporal relationship between the increase of COVID-19 incidence with a decrease in the number of LTs performed, and a subsequent spike in LT waitlist mortality rates. This trend was further verified at the regional level by stratifying national data by UNOS region along with region-reported COVID incidence rates.

The highly intricate impact of the COVID-19 pandemic on ICU capacity, hospital resources, elective surgeries, traveling to out-of-state donor centers, and potential deleterious effects of the virus on LT recipients curtailed the number of LTs performed and provided a natural experiment on the impact of liver transplantation on mortality prevention (11). Our results revealed that the decline in LTs performed in the United States was corelated with an increase in LT waitlist mortality in many UNOS regions, especially those with high-volume LT centers such as 3, 5, 7, and 10 who normally shoulder the LT clinical demand. This finding is especially concerning on an international scale since many countries in Europe decreased their LT activity by over 90%, with waitlist mortality ramifications yet to be reported (2, 8, 12, 13).

The transplant community in the United States began to recover by week 15 of 2020, taking 5 weeks to resume operating at full capacity by week 20. Subsequently by week 20, LT waitlist mortality had decreased to its pre-COVID baseline. The recovery suggests that after the initial COVID-19 surge, hospitals took measures to resume surgeries and once again allocate resources to clinical areas outside of COVID-19 (14–17).

The pandemic further exposed the already known variation in LT practice in the United States between UNOS regions and LT centers (18, 19). LT candidates were victim to two new forces outsides of their control during the pandemic—their region's prevention response to COVID-19 and the subsequent healthcare burden on hospital infrastructure, including transplant personnel. Regions with high initial COVID-19 rates in 2020 were forced reduce LT activity, including listing new patients, which unfortunately occurred in many high-volume centers with serious waitlist mortality consequences. Variations in social distancing practices, travel bans in and out of regions, and the availability of COVID-19 testing also likely contributed to the discrepancies in LT waitlist mortality seen between different UNOS regions (2, 20). As the United States enters another COVID-19 surge, it becomes imperative for transplant centers to continue liver transplantation as our data verifies waitlisted candidates cannot wait for the surge to pass.

This study was not without limitations. First, OPTN data is self-reported and has variations in reporting consistency between transplant centers. This data also reports outcomes on a regional level in the STAR file but not at a donation service area (DSA) level, which would result in a more granular analysis (21). Additionally, each transplant center moved forward with an individualized approach to performing LT during the pandemic, which was influenced by institutional-specific factors which are not evaluated by this study. The American Society of Transplant Surgeons (ASTS) released suggested guidelines based on available data, but encouraged each center to develop their own protocols based on resources, the prevalence of COVID-19, and their patient's needs.

Despite these limitations, there are key takeaways from this work that can be implemented in future pandemics. At the time of this writing, <20% of Americans are fully vaccinated against COVID-19 and incidence rates are again rising in 28 out of 50 states (10), forcing some states such as Michigan to begin restricting elective surgeries. Additionally, there are over 16,000 COVID-19 variant cases in the U.S. as well (10). These recent trends indicate that we have not overcome the pandemic, and must learn from the initial COVID-19 surge to better protect our liver transplant waitlist population. Specifically, there needs to be a standardization among transplant centers in reporting COVID-19 cases, ICU availability, and waitlist inactivations to maintain equity in access to LT across the United States. Second, we have seen that brief pauses in liver transplant activity lead to rapid increases in waitlist mortality, so operating room, ICU, and clinical resources need to be pre-allocated for the uninterrupted practice of liver transplantation through future pandemic surges. Finally, our results showed a rapid decrease in number of deceased donors during the initial pandemic surge, partly due to travel bans between states. In the event that future travel bans are implemented, transplant centers need to ensure that these restrictions do not interfere with the procurement of organs in acuity circles.

In conclusion, this study reinforces the necessity of liver transplantation as an essential, non-elective health service due to the clinical severity of LT candidates and the lack of a temporizing option, which results in high mortality rates for these patients without transplantation. The interruptions in LT activity due to COVID-19 correlated with rapid rises in mortality and a decline in new waitlist additions. Given our initial experience with COVID-19, the transplant community can utilize this data to advocate for and prepare to continue the uninterrupted practice of liver transplantation should another COVID surge (with new variants) or another pandemic arise.

## Data Availability Statement

Publicly available datasets were analyzed in this study. This data can be found here: https://optn.transplant.hrsa.gov/data/request-data/.

## Author Contributions

QY, OH, and TC wrote the manuscript. QY ran the statistical analysis and wrote the methods. OH, QY, TC, and JM participated in the critical revision of the manuscript for intellectual content. All authors contributed to the preparation of the manuscript.

## Conflict of Interest

The authors declare that the research was conducted in the absence of any commercial or financial relationships that could be construed as a potential conflict of interest.

## Abbreviations

ASTS: American Society of Transplant Surgeons
DBD: donation after brain death
DCD: donation after circulatory death
DSA: donation service area
ICU: intensive care unit
LT: liver transplant
MELD: model for end-stage liver disease
OPTN: Organ Procurement and Transplantation Network
UNOS: United Network for Organ Sharing.
