# Supplementary figures and images for: The Heterogenous Effect of COVID-19 on Liver Transplantation Activity and Waitlist Mortality in the United States
Source: Front Surg. 2021 May 18;8:669129. doi: 10.3389/fsurg.2021.669129 (PMC8171664; doi:10.3389/fsurg.2021.669129)

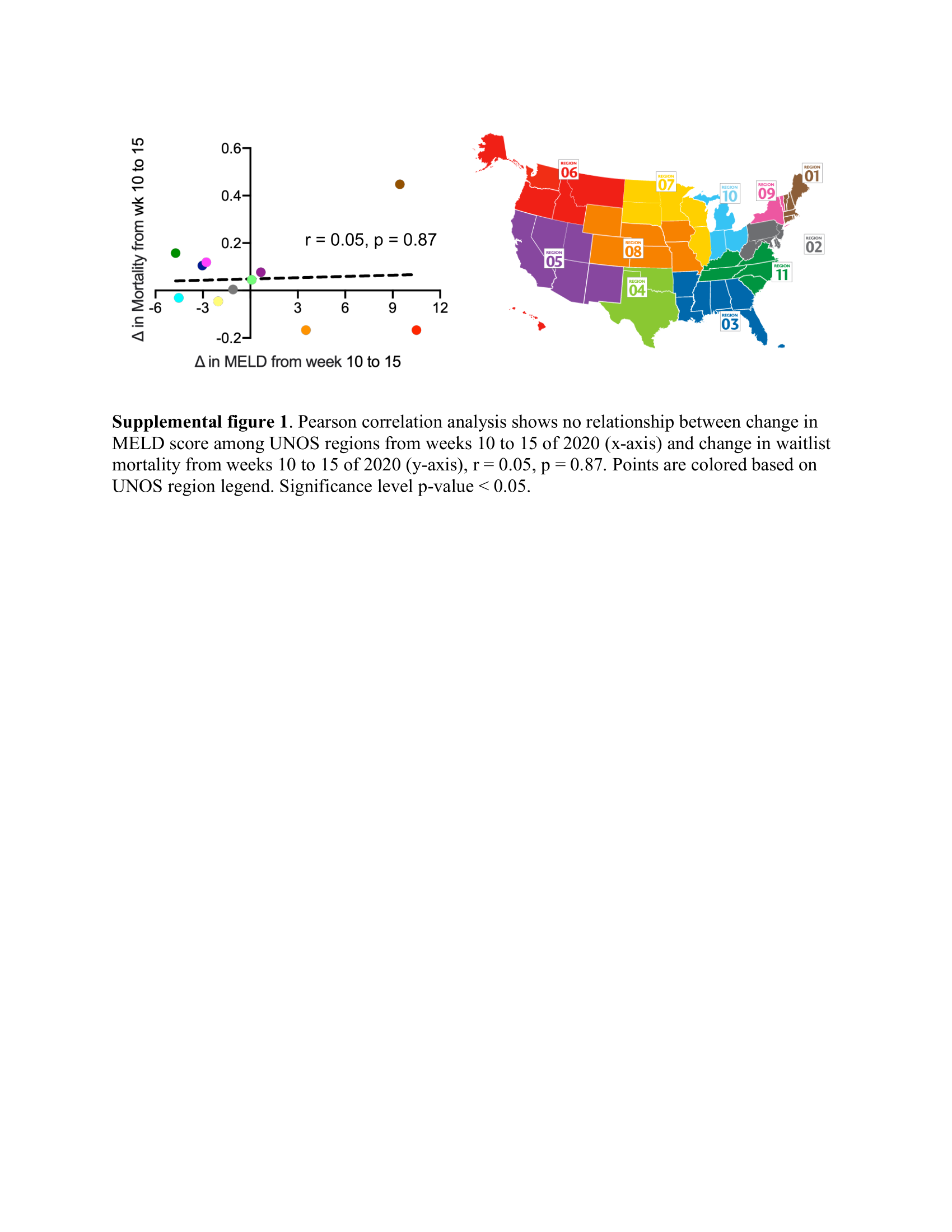

Supplement: Supplementary file 1 [file Image_1.TIFF]
